# Supplementary material for: Cardiac neural crest contributes to cardiomyocytes in amniotes and heart regeneration in zebrafish
Source: eLife. 2019 Aug 8;8:e47929. doi: 10.7554/eLife.47929 (PMC6721792; doi:10.7554/eLife.47929)
Supplement: Supplementary file 1. — (a, b) Quantification of cardiac neural crest contribution to the heart in chick and mouse.Table presents virally labeled cardiac neural crest derivatives at MNC (migratory neural crest), CN-IX (cranial nerve nine), PAA (pharyngeal arch arteries), APS (aorticopulmonary septum), MYO (myocardium of ventricle) and IVS (interventricular septum) at day 1–6 and day 10 post injection in chick. The bottom part presents number of Wnt1+ cells in E15.5 Wnt1-Cre mouse. Percentage in parentheses represents the proportion of the population among all NC-derived cells in cardiovascular structure (including MYO, APS, and IVS). % Neural crest contribution to ventricle, the proportion of Wnt1+ cells (including MYO and IVS) among all cells in the ventricle is about 16.8%. Supplementary file 1b shows the raw data of each embryo from which data in Supplementary file 1a was generated. (c) Quantification of sox10:eGFP+ cells in the apex during zebrafish heart regeneration. Average number of Sox10-eGFP+ cells per 2 × 10^5^ µm2 in one section through the middle of the apex of 7dpa (n = 3) and 21 dpa (n = 3) hearts after resection. Standard deviation is presented in parentheses next to the cell number. GFP expression was negligible in sham operated hearts (n = 3) at the same time points. [file elife-47929-supp1.pdf]

Supplementary File 1a
Quantification of cardiac neural crest contribution to the heart in chick and mouse

					Cardiovascular structure		
	Stage	MNC	CN-IX	PAA	APS (%)	MYO (%)	IVS (%)
Chick retroviral lineage tracing	Day 1	15	12	22			
	Day 2	38	36	199			
	Day 3			112	16	7	
	Day 4			64	65 (73.9)	11 (12.5)	12(13.6)
	Day 5			270	197 (90.4)	6 (2.8)	15 (6.8)
	Day 6			199	80 (84.2)	7 (7.4)	8 (8.4)
	Day 10				201 (84.5)	12 (5)	25 (10.5)
Wnt1-ZsGreen	E 15.5				169 (58.1)	95 (32.6)	27 (9.3)

Abbreviations: MNC, migratory neural crest; CN-IX, cranial nerve nine; PAA, pharyngeal arch arteries; APS, aorticopulmonary septum; MYO, myocardium of ventricle; IVS, interventricular septum.

Supplementary File 1b
Quantification of cardiac neural crest contribution to the heart in chick and mouse

	MNC	CN-IX	PAA	APS	MYO	IVS
D1 E1	7	11	20			
D1 E2	31	14	22			
D1 E3	5	14	26			
D1 E4	10	15	19			
D1 E5	24	6	22			
D2 E1	45	49	36			
D2 E2	63	73	195			
D2 E3	21	23	53			
D2 E4	27	15	517			
D2 E5	34	21	190			
D3 E1			82	9	12	
D3 E2			88	24	7	
D3 E3			49	17	7	
D3 E4			228	15	1	
D4 E1			46	129	20	7
D4 E2			49	11	7	0
D4 E3			44	35	11	26
D4 E4			115	85	6	15
D5 E1			265	446	7	8
D5 E2			270	144	7	3
D5 E3			227	97	6	18
D5 E4			316	99	5	31
D6 E1			106	48	6	16
D6 E2			211	117	9	8
D6 E3			282	61	5	0
D6 E4			198	92	6	9
D10 E1				77	7	32
D10 E2				422	18	26
D10 E3				51	13	23
D10 E4				254	8	19
E15.5 Wnt1-Zsgreen E1				146	86	32
E15.5 Wnt1-Zsgreen E2				149	80	33
E15.5 Wnt1-Zsgreen E3				212	145	35
E15.5 Wnt1-Zsgreen E4				170	70	8

Abbreviations: MNC, migratory neural crest; CN-IX, cranial nerve nine; PAA, pharyngeal arch arteries; APS, aorticopulmonary septum; MYO, myocardium of ventricle; IVS, interventricular septum.

Supplementary File 1c

Quantification of *sox10:eGFP+* cells in the apex during zebrafish heart regeneration

	7 dpa	21 dpa	Sham
sox10:eGFP (cell/ $2x10^5$ μm^2)	17 (± 6.7)	29 (± 4.0)	2 (± 1.2)

Abbreviation: dpa, days post amputation.
